# Association between CYP17A1 rs743572 polymorphism and cancer risk: A meta-analysis

**DOI:** 10.1371/journal.pone.0326843

**Published:** 2025-06-25

**Authors:** Bin Wang, Zhumin Cao, Ying Li, Hua Zou

**Affiliations:** 1 Department of Oncology, the Seventh People’s Hospital of Chongqing (Affiliated Central Hospital of Chongqing University of Technology), Chongqing, China; 2 Vascular interventional department, the Seventh People’s Hospital of Chongqing (Affiliated Central Hospital of Chongqing University of Technology), Chongqing, China; 3 Department of Pharmacy, Chongqing Seventh People’s Hospital(Affiliated Central Hospital of Chongqing University of Technology), Chongqing, China; 4 Cancer Center, Daping Hospital, Army military Medical University, Chongqing, China; Sukh Sagar Medical College and Hospital, INDIA

## Abstract

The role of the CYP17A1 gene’s rs743572 polymorphism in cancer susceptibility has been a subject of extensive investigation, yet existing evidence remains inconclusive. In this meta-analysis, we systematically reviewed and synthesized data from 29 studies to assess the CYP17A1 rs743572 polymorphism’s relationship with cancer susceptibility. We strictly searched on EMBASE, PubMed, and Web of Science databases and explored rs743572 polymorphism’s association with cancer risks according to search strategy, enrolling 29 studies (13,767 cases and 17,441 controls). rs743572 was markedly related to enhanced cancer susceptibility risk; FPRP and TSA analyses were employed for confirmation. According to cancer type-based stratified analyses, rs743572 exhibited a notable association with bladder cancer, breast cancer, non-Hodgkin lymphoma, and hepatocellular cancer. In conclusion, systematic meta-analysis suggests a significant role for the rs743572 polymorphism in cancer pathogenesis, with particular prominence observed in bladder cancer, breast cancer, non-Hodgkin lymphoma, and hepatocellular cancer.

## 1. Introduction

Cancer remarkably affect public health worldwide. As per the GLOBOCAN 2020 report, the anticipated figures for 2020 indicate a staggering 19.3 million new cancer cases and 10.0 million deaths globally [1]. A multitude of environment- and gene-related factors contribute to the emergence of cancer, though the underlying mechanisms remain incompletely elucidated [2].

Cytochrome P450 enzymes are crucial for human health, affecting various endogenous and exogenous components’ metabolism [3]. Among these enzymes, CYP17A1, belonging to the cytochrome P450 family, is particularly pivotal for human health and its association with cancer [4]. CYP17A1 is involved in steroidogenesis, a crucial process of steroid hormones biosynthesis [5]. Specifically, CYP17A1 catalyzes the 17α-hydroxylation of pregnenolone and progesterone, producing intermediates that serve as precursors for synthesizing androgens and estrogens [5]. Dysregulated steroid hormone production, often mediated by CYP17A1, closely participates in the pathogenesis of multiple cancers: prostate cancer, hormone-sensitive breast cancer, etc. [6]. In prostate cancer, for instance, CYP17A1 is central for androgen biosynthesis, contributing to producing testosterone and dihydrotestosterone, which drive the growth of prostate tumors [6]. As a result, CYP17A1 has become a target for therapeutic intervention in prostate cancer treatment. Understanding the intricate relationship between cytochrome P450 enzymes, particularly CYP17A1, and cancer provides valuable insights into the development of targeted therapies aimed at modulating steroid hormone levels. By unraveling the molecular mechanisms underlying these processes, researchers strive to devise innovative strategies to mitigate the impact of dysregulated CYP17A1 activity on cancer progression, contributing to advancements in cancer treatment and improving overall human health.

In recent times, genome-wide association studies pinpointed a notable relationship between CYP17A1 polymorphisms and steroid hormone levels [7]. Notably, the CYP17A1 rs743572 polymorphism has been implicated in multiple cancers, spanning organs such as breast, lung, prostate, liver, etc. However, existing findings exhibit inconsistencies, likely stemming from limited sample sizes. To provide a more comprehensive understanding of this association, a series of analyses were conducted.

## 2. Materials and methods

### 2.1. Research retrieval

EMBASE, PubMed, and Web of Science were retrieved, covering studies up to December 20, 2023, to identify relevant research investigating the association between the CYP17A1 rs743572 polymorphism and cancer risks, as outlined in the search strategy detailed in Table 1. Four authors independently executed the search and screened the results.

**Table 1:** The detailed search strategies of lCYP17A1 rs743572 polymorphism’s association with cancer susceptibility.

Source	Search strategy	
Pubmed	#1: Polymorphism, genetic #2: Polymorphism^*^ #3: SNP #4: Single nucleotide polymorphism #5: Variant #6: Mutation #7: #1 OR #2 OR #3 OR #4 OR #5 OR #6 #8: rs743572 #9: CYP17A1 #10: #8 AND #9 #11: Neoplasms #12: Cancer #13: Carcino^*^ #14: #11 OR #12 OR #13 #15: #7 AND #10 AND #14	((Polymorphism, genetic) OR Polymorphism^*^ OR SNP OR (Single nucleotide polymorphism) OR Variant OR Mutation) AND (rs743572 AND CYP17A1) AND (Neoplasms OR Cancer OR Carcino^*^))
Embase	#1: ‘neoplasm’/exp #2: cancer #3: tumor #4: carcinoma #5: carcinogenesis #6: #1 OR #2 OR #3 OR #4 OR #5 #7: rs743572 #8: CYP17A1 #9: #7 AND #8 #10: ‘single nucleotide polymorphism’/exp #11: SNP #12: polymorphism #13: variant #14: mutation #15: #10 OR #11 OR #12 OR #13 OR #14 #16: #6 AND #9 AND #15	((‘neoplasm’/exp OR cancer OR tumor OR carcinoma OR carcinogenesis) AND (rs743572 AND CYP17A1) AND (‘single nucleotide polymorphism’/exp OR SNP OR polymorphism OR variant OR mutation))

### 2.2. Inclusion and exclusion criteria

The studies included in our analysis adhered to the following inclusion criteria: (A) Human-based research; (B) Case-control or cohort study design; (C) Availability of valid data; (D) Investigation into CYP17A1 rs743572 polymorphism’s relationship with cancer; (E) Controls meeting Hardy-Weinberg Equilibrium (HWE), with *P* > 0.05. *P *> 0.05 represents genetic equilibrium in the population [8]. Additionally, the exclusion criteria was: (A) Case-only/non-cancer subject-only research; (B) Repetitive research; (C) Assembly abstracts.

### 2.3. Data screening

Valid data were screened independently by two researchers, encompassing first author’s name, publication date, origin country, ethnicity, cancer type, and control source, as well as cases and controls’ numbers in each study.

### 2.4. Quality evaluation

Each study’s quality was independently evaluated by two researchers with the quality assessment criteria outlined in S1 Table, as previously described [9]. The following aspects were involved: cancer case ascertainment (0–2), case representation (0–2), control representation (0–3), control selection (0–2), genotyping examination (0–2), adherence to HWE (0–1), and total sample size (0–3). Total scores ranged from 0 to 15, with studies scoring > 9 points categorized as high quality.

### 2.5. Statistical analysis

Statistical analysis was conducted using Stata software (Version 12.0, StataCorp, College Station, TX, USA). The association between the CYP17A1 rs743572 polymorphism and cancer risks was assessed using odds ratio (OR) and 95% confidence interval (CI). Dominant, recessive, homozygote, heterozygote, and allele models were employed for analysis. Cochrane Q-test and P-values were applied for heterogeneity evaluation: random-effects model with *P *≤ 0.10 or *I*^*2 *^≥ 50%; fixed-effects model otherwise. Information was stratified according to ethnicity, control source, and cancer type. Publication bias was assessed through Begg’s test and Egger’s test. Study reliability was evaluated by analyzing sensitivity. *P* < 0.05 represented statistical significance. False positive report probability (FPRP) analysis was carried out as previously mentioned [10], which assumed 0.1 as the prior probability, with an FPRP threshold of 0.2 considered noteworthy [11]. To address potential biases from sparse data that may impact meta-analyses, trial sequential analysis (TSA) was conducted using the Copenhagen Trial Unit’s software (Denmark, 2011). The study parameters included overall type–I error (5%), test power (80%), and relative risk reduction (20%).

## 3. Results

### 3.1. Study selection

**Fig 1:**
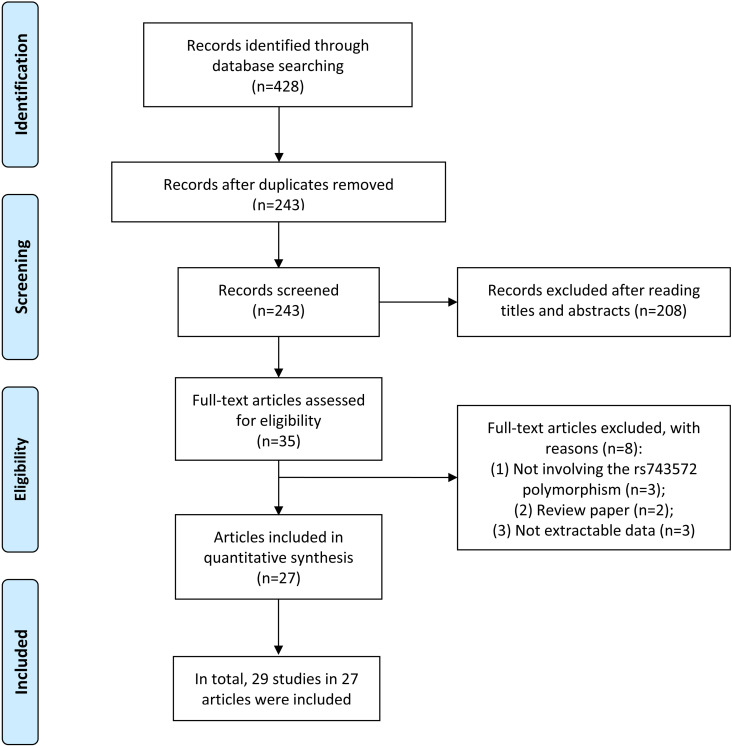
Study identification and selection.

Our search strategy yielded 428 articles. Following evaluating titles and abstracts, 35 articles met our inclusion criteria. Upon a thorough examination of the full texts, 8 articles were excluded. This exclusion comprised three articles that did not address the association between CYP17A1 rs743572 polymorphism and cancer susceptibility, two review articles, and three that lacked detailed genotyping data. Ultimately, 27 articles, encompassing 29 studies (13,767 cases; 17,441 controls) were included [12–38]. The screening process is illustrated in Fig 1.

Broadly, the meta-analysis involved 21 studies with Caucasian populations and 8 studies with Asian populations. Out of the total, 14 studies focused on the impact of CYP17A1 rs743572 polymorphism in breast cancer, 1 in bladder cancer, 2 in colorectal cancer, 2 in pancreatic cancer, 6 in prostate cancer, 1 in endometrial cancer, 1 in non-Hodgkin lymphoma, 1 in hepatocellular cancer, and 1 in non-small cell lung cancer (Table 2).

**Table 2:** Details of included studies.

First Author	Year	Ethnicity	Cancer type	Control source	Genotyping method	Cases (CC/TC/TT)	Controls (CC/TC/TT)	HWE	Score
Bethke	2007	Caucasian	Colorectal cancer	PB	Illumina	995/1192/374	1045/1234/416	0.102	12
Chattopadhyay	2014	Asian	Breast cancer	PB	PCR-RFLP	230/116/14	260/93/7	0.692	10
Duell	2010	Caucasian	Pancreatic cancer	PB	MassARRAY	101/113/32	287/372/141	0.283	7
Farzaneh	2016	Asian	Breast cancer	HB	PCR-RFLP	32/70/22	27/56/17	0.189	6
Ghisari	2014	Caucasian	Breast cancer	HB	TaqMan	24/57/32	12/12/6	0.882	7
Ghisari	2017	Caucasian	Breast cancer	HB	TaqMan	52/65/23	71/88/33	0.524	10
He	2022	Asian	Breast cancer	HB	MassARRAY	119/131/52	183/89/30	0.128	9
Hussain	2016	Asian	Pancreatic cancer	HB	PCR-RFLP	72/122/61	119/154/47	0.805	7
Iwasaki-1	2010	Asian	Breast cancer	HB	TaqMan	111/189/88	122/182/84	0.299	10
Iwasaki-2	2010	Caucasian	Breast cancer	HB	TaqMan	17/48/13	23/33/23	0.144	10
Iwasaki-3	2010	Caucasian	Breast cancer	HB	TaqMan	135/185/59	130/200/49	0.139	11
Karakus	2015	Caucasian	Breast cancer	HB	PCR-RFLP	102/79/18	104/78/15	0.143	8
Martinez-Gonzalez	2020	Caucasian	Prostate cancer	HB	TaqMan	89/67/5	95/61/6	0.317	7
Olson	2008	Caucasian	Endometrial cancer	PB	PCR-RFLP	122/207/71	148/210/59	0.297	9
Martin-Way	2022	Caucasian	Bladder cancer	HB	TaqMan	58/43/6	78/21/0	0.589	10
MARIE-GENICA Consortium on Genetic Susceptibility for Menopausal Hormone Therapy Related Breast Cancer Risk	2010	Caucasian	Breast cancer	PB	MassARRAY	1043/1573/529	1834/2712/941	0.254	12
Reding	2012	Caucasian	Breast cancer	PB	PCR-RFLP	241/356/50	234/315/100	0.722	13
Risio	2011	Caucasian	Prostate cancer	PB	SNPlex assay	142/62/28	45/20/4	0.388	11
Rizzolo	2019	Caucasian	Breast cancer	HB	TaqMan	190/301/106	347/489/186	0.550	11
Robles-Fernandez	2017	Caucasian	Prostate cancer	HB	TaqMan	67/66/23	53/82/20	0.178	9
Rudolph	2011	Caucasian	Colorectal cancer	HB	PCR-RFLP	213/355/115	252/326/103	0.885	11
Sakoda	2008	Caucasian	Breast cancer	PB	PCR-RFLP	216/297/102	298/441/138	0.232	7
Skibola	2005	Caucasian	Non-Hodgkin lymphoma	PB	SNPlex assay	237/274/93	308/366/83	0.095	14
Song	2016	Asian	Prostate cancer	HB	PCR-RFLP	38/76/62	43/71/54	0.050	10
Tang	2018	Caucasian	Prostate cancer	HB	SNPlex assay	219/323/73	189/259/70	0.205	7
TÜZÜNER	2010	Caucasian	Breast cancer	HB	PCR-RFLP	18/27/10	38/44/9	0.466	9
Mattia	2017	Caucasian	Hepatocellular cancer	HB	TaqMan	39/88/40	72/84/33	0.325	10
Zhang	2013	Asian	Non-small cell lung cancer	HB	MassARRAY	59/108/37	72/91/37	0.386	11
Wu	2020	Asian	Prostate cancer	HB	TaqMan	14/33/11	25/27/6	0.744	7

Note: PB: publication-based controls; HB: hospital-based controls; C: wild type; T: mutated type.

### 3.2. Meta-analysis of rs743572

**Fig 2:**
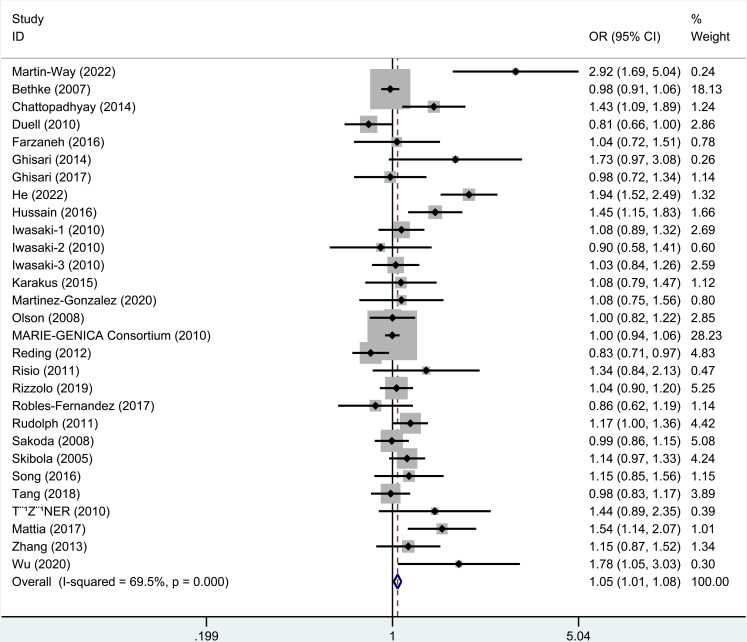
Meta-analysis for rs743572 polymorphism’s association with cancer risk (allele model). Each study is represented by a square (point estimate) and a horizontal line (95% confidence interval [CI]). The size of the square corresponds to the study’s weight in the meta-analysis. The diamond summarizes the overall pooled odds ratio (OR) and 95% CI. Significant associations (OR > 1) indicate increased cancer risk with the allele.

**Fig 3:**
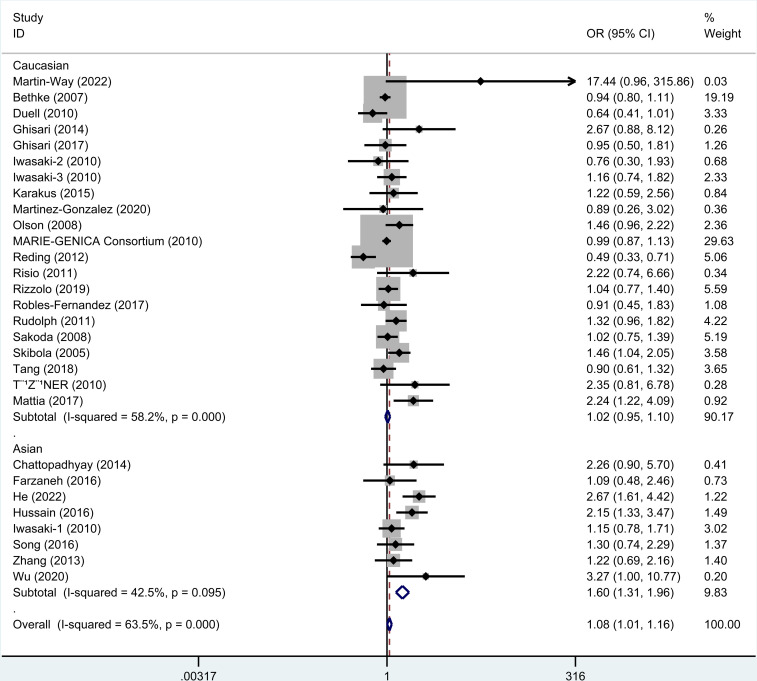
Meta-analysis for rs743572 polymorphism’s association with cancer risk stratified by ethnicity (homozygote model). Subgroup analyses for Caucasian and Asian populations are shown. Significant associations were found in Asians (dominant, OR=1.50, 95% CI = 1.30-1.72; recessive, OR=1.32, 95% CI = 1.11-1.58; homozygote, OR=1.60, 95% CI = 1.31-1.96; heterozygote, OR=1.43, 95% CI = 1.24-1.66; Allele, OR=1.32, 95% CI = 1.20-1.46) and Caucasian populations (heterozygote, OR=1.05, 95% CI = 1.00-1.11).

**Fig 4:**
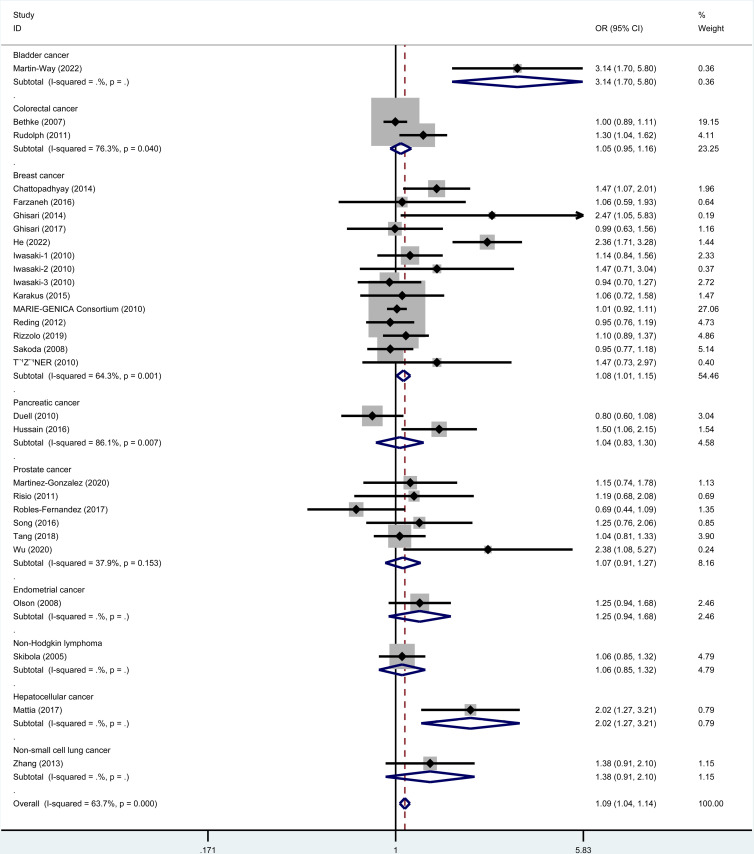
Meta-analysis for rs743572 polymorphism’s association with cancer risk stratified by cancer type (dominant model). Stratification by cancer type revealed significant risk increases for bladder cancer (OR = 3.14, 95% CI = 1.70–5.80), breast cancer (OR = 1.08, 95% CI = 1.01–1.15), and hepatocellular carcinoma (OR = 2.02, 95% CI = 1.27–3.21).

**Fig 5:**
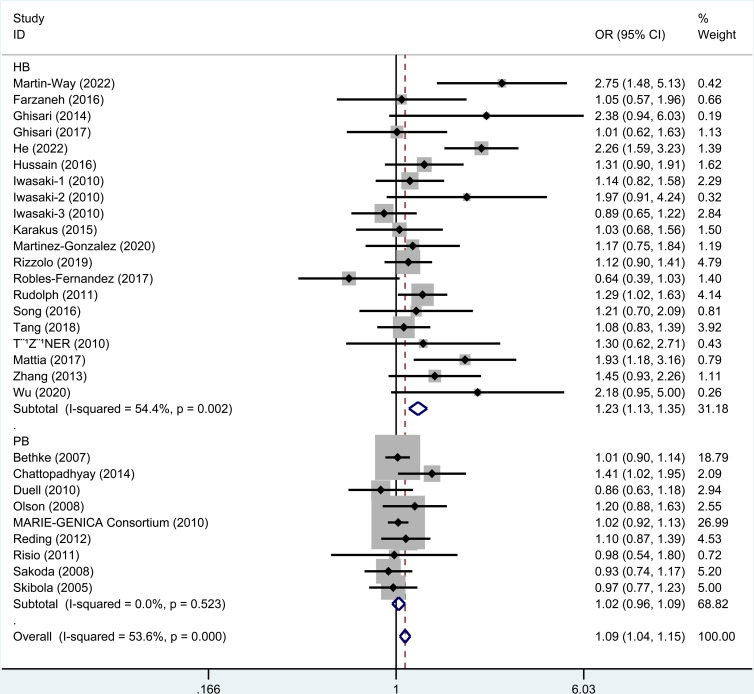
Meta-analysis for rs743572 polymorphism’s association with cancer risk stratified by control source (heterozygote model). Hospital-based (HB) controls showed a significant association (OR = 1.23, 95% CI = 1.13–1.35; I² = 54.4%), whereas population-based (PB) controls did not (OR = 1.02, 95% CI = 0.96–1.09; I² = 0.0%). The overall effect remained significant (OR = 1.09, 95% CI = 1.04–1.15; I² = 53.6%), suggesting potential selection bias in HB studies.

As revealed by the results (Table 3), rs743572 polymorphism notably elevated cancer risk in dominant (OR=1.09, 95% CI = 1.04–1.14), heterozygote (OR=1.09, 95% CI = 1.04–1.15), homozygote (OR=1.08, 95% CI = 1.01–1.16), and allele (OR=1.05, 95% CI = 1.01–1.08, Fig 2) models. When studies were stratified by ethnicity, significant associations were found in Asians (dominant, OR=1.50, 95% CI = 1.30–1.72; recessive, OR=1.32, 95% CI = 1.11–1.58; homozygote, OR=1.60, 95% CI = 1.31–1.96, Fig 3; heterozygote, OR=1.43, 95% CI = 1.24–1.66; Allele, OR=1.32, 95% CI = 1.20–1.46) and Caucasian populations (heterozygote, OR=1.05, 95% CI = 1.00–1.11). When studies were stratified by cancer type, rs743572 polymorphism increased bladder cancer risk (dominant, OR=3.14, 95% CI = 1.70–5.80, Fig 4; heterozygote, OR=2.75, 95% CI = 1.48–5.13; Allele, OR=2.92, 95% CI = 1.69–5.04), breast cancer (dominant, OR=1.08, 95% CI = 1.01–1.15, Fig 4; heterozygote, OR=1.08, 95% CI = 1.01–1.16), non-Hodgkin lymphoma (recessive, OR=1.48, 95% CI = 1.08–2.03; homozygote, OR=1.46, 95% CI = 1.04–2.05), and hepatocellular cancer (dominant, OR=2.02, 95% CI = 1.27–3.21, Fig 4; homozygote, OR=2.24, 95% CI = 1.22–4.09; heterozygote, OR=1.93, 95% CI = 1.18–3.16; Allele, OR=1.54, 95% CI = 1.14–2.07). Additionally, a remarkable association between rs743572 polymorphism and elevated cancer risk was noted in studies with publication-based controls and hospital-based controls (Fig 5). According to Table 4, With a prior probability assumed as 0.1, associations with statistical significance were noteworthy (FPRP< 0.2) for overall cancer risk (dominant, heterozygote, homozygote, and allele models), Asians (dominant, recessive, homozygote, heterozygote, and allele models), Caucasian populations (heterozygote model), bladder cancer (dominant, heterozygote, and allele models), breast cancer (dominant and heterozygote models), non-Hodgkin lymphoma (recessive and homozygote models), hepatocellular cancer (dominant, homozygote, heterozygote, and allele models) subgroups (Table 4).

**Table 3:** Meta-analysis of CYP17A1 rs743572 polymorphism’s association with cancer risk.

Variables	No. of study	No. of cases/controls	Dominant model (TT + TC *vs.* CC)	Recessive model (TT *vs.* TC + CC)	Homozygote model (TT *vs.* CC)	Heterozygote model (TC *vs.* CC)	Allele model (T *vs.* C)
			OR (95%CI)/*I*^*2*^%	OR (95%CI)/*I*^*2*^%	OR (95%CI)/*I*^*2*^%	OR (95%CI)/*I*^*2*^%	OR (95%CI)/*I*^*2*^%
Overall	29	13773/17661	**1.09 (1.04, 1.14)/63.7**	1.03 (0.97, 1.10)/56.8	**1.08 (1.01, 1.16)/63.5**	**1.09 (1.04, 1.15)/53.6**	**1.05 (1.01, 1.08)/69.5**
Caucasian	21	11910/15761	1.04 (0.99, 1.10)/50.9	0.99 (0.93, 1.06)/56.9	1.02 (0.95, 1.10)/58.2	**1.05 (1.00, 1.11)/41.6**	1.01 (0.98, 1.05)/57.6
Asian	8	1683/1900	**1.50 (1.30, 1.72)/48.9**	**1.32 (1.11, 1.58)/27.1**	**1.60 (1.31, 1.96)/42.5**	**1.43 (1.24, 1.66)/34.9**	**1.32 (1.20, 1.46)/62.7**
Bladder cancer	1	99/107	**3.14 (1.70, 5.80)/-**	12.74 (0.71, 229.24)/-	17.44 (0.96, 315.86)/-	**2.75 (1.48, 5.13)/-**	**2.92 (1.69, 5.04)/-**
Colorectal cancer	2	3244/3376	1.05 (0.95, 1.16)/76.3	0.98 (0.85, 1.12)/25.1	1.01 (0.87, 1.17)/69.7	1.06 (0.96, 1.18)/68.2	1.02 (0.95, 1.09)/74.9
Breast cancer	14	7059/10236	**1.08 (1.01, 1.15)/64.3**	0.99 (0.91, 1.07)/62.9	1.03 (0.94, 1.13)/65.7	**1.08 (1.01, 1.16)/55.8**	1.03 (0.99, 1.08)/71.2
Pancreatic cancer	2	563/1284	1.04 (0.83, 1.30)/86.1	1.10 (0.83, 1.46)/90.1	1.11 (0.81, 1.52)/92.3	1.02 (0.80, 1.30)/64.3	1.05 (0.90, 1.22)/92.3
Prostate cancer	6	1398/1130	1.07 (0.91, 1.27)/37.9	1.07 (0.85, 1.35)/0	1.11 (0.85, 1.44)/24.1	1.06 (0.89, 1.26)/34.7	1.05 (0.94, 1.19)/30.9
Endometrial cancer	1	417/400	1.25 (0.94, 1.68)/-	1.31 (0.90, 1.91)/-	1.46 (0.96, 2.22)/-	1.20 (0.88, 1.63)/-	1.00 (0.82, 1.22)/-
Non-Hodgkin lymphoma	1	604/757	1.06 (0.85, 1.32)/-	**1.48 (1.08, 2.03)/-**	**1.46 (1.04, 2.05)/-**	0.97 (0.77, 1.23)/-	1.14 (0.97, 1.33)/-
Hepatocellular cancer	1	189/167	**2.02 (1.27, 3.21)/-**	1.49 (0.89, 2.50)/-	**2.24 (1.22, 4.09)/-**	**1.93 (1.18, 3.16)/-**	**1.54 (1.14, 2.07)/-**
Non-small cell lung cancer	1	200/204	1.38 (0.91, 2.10)/-	0.98 (0.59, 1.62)/-	1.22 (0.69, 2.16)/-	1.45 (0.93, 2.26)/-	1.15 (0.87, 1.52)/-
HB	20	4884/5403	**1.25 (1.15, 1.36)/61.9**	**1.15 (1.03, 1.28)/25.8**	**1.30 (1.15, 1.46)/43.1**	**1.23 (1.13, 1.35)/54.4**	**1.16 (1.09, 1.23)/64.7**
PB	9	8889/12258	1.02 (0.96, 1.08)/27.4	0.97 (0.90, 1.05)/76.2	0.98 (0.90, 1.07)/73.7	1.02 (0.96, 1.09)/0	0.99 (0.95, 1.03)/59.8
High quality (> 9)	16	10478/13404	**1.07 (1.02, 1.13)/55.2**	0.99 (0.93, 1.07)/62.6	1.04 (0.96, 1.12)/61.6	**1.08 (1.02, 1.14)/46.5**	1.03 (0.99, 1.07)/65.3
Low quality (≤ 9)	13	3295/4257	**1.15 (1.04, 1.26)/71.7**	**1.16 (1.01, 1.33)/42.0**	**1.23 (1.06, 1.42)/64.5**	**1.12 (1.01, 1.24)/62.4**	**1.09 (1.02, 1.17)/74.5**

**Table 4:** FPRP values for CYP17A1 rs743572 polymorphism’s association with cancer risk.

Significant association	OR (95%CI)	Prior probability				
		0.25	0.1	0.01	0.001	0.0001
**Dominant model (TT + TC *vs.* CC)**						
Overall	1.09 (1.04, 1.14)	**0.036**	**0.147**	0.342	0.937	0.998
Asian	1.50 (1.30, 1.72)	**0.011**	**0.089**	**0.112**	**0.145**	0.289
Bladder cancer	3.14 (1.70, 5.80)	**0.003**	**0.009**	**0.013**	**0.056**	**0.132**
Breast cancer	1.08 (1.01, 1.15)	**0.046**	**0.153**	0.267	0.463	0.895
Hepatocellular cancer	2.02 (1.27, 3.21)	**0.024**	**0.087**	**0.133**	**0.185**	0.965
HB	1.25 (1.15, 1.36)	**0.005**	**0.012**	**0.114**	0.565	0.927
High quality (> 9)	1.07 (1.02, 1.13)	**0.026**	**0.174**	0.643	0.864	0.995
Low quality (≤ 9)	1.15 (1.04, 1.26)	**0.008**	**0.024**	**0.165**	0.742	0.996
**Recessive model (TT *vs.* TC + CC)**						
Asian	1.32 (1.11, 1.58)	**0.003**	**0.016**	**0.072**	**0.146**	0.218
Non-Hodgkin lymphoma	1.48 (1.08, 2.03)	**0.006**	**0.028**	**0.053**	**0.113**	**0.186**
HB	1.15 (1.03, 1.28)	**0.009**	**0.017**	**0.113**	0.651	0.984
Low quality (≤ 9)	1.16 (1.01, 1.33)	**0.035**	**0.139**	0.237	0.581	0.968
**Homozygote model (TT *vs.* CC)**						
Overall	1.08 (1.01, 1.16)	**0.077**	**0.187**	0.852	0.982	0.999
Asian	1.60 (1.31, 1.96)	**0.003**	**0.009**	**0.067**	**0.136**	**0.178**
Non-Hodgkin lymphoma	1.46 (1.04, 2.05)	**0.005**	**0.018**	**0.097**	**0.134**	0.265
Hepatocellular cancer	2.24 (1.22, 4.09)	**0.001**	**0.007**	**0.015**	**0.083**	**0.117**
HB	1.30 (1.15, 1.46)	**0.009**	**0.045**	0.209	0.454	0.935
Low quality (≤ 9)	1.23 (1.06, 1.42)	**0.015**	**0.189**	0.539	0.878	0.996
**Heterozygote model (TC *vs.* CC)**						
Overall	1.09 (1.04, 1.15)	**0.011**	**0.182**	0.643	0.783	0.999
Caucasian	1.05 (1.00, 1.11)	**0.018**	**0.190**	0.788	0.896	0.997
Asian	1.43 (1.24, 1.66)	**0.007**	**0.013**	**0.145**	0.411	0.832
Bladder cancer	2.75 (1.48, 5.13)	**0.003**	**0.018**	**0.069**	**0.116**	**0.158**
Breast cancer	1.08 (1.01, 1.16)	**0.025**	**0.146**	0.367	0.686	0.997
Hepatocellular cancer	1.93 (1.18, 3.16)	**0.003**	**0.011**	**0.102**	**0.164**	0.227
HB	1.23 (1.13, 1.35)	**0.025**	**0.170**	0.232	0.567	0.916
High quality (> 9)	1.08 (1.02, 1.14)	**0.049**	**0.137**	0.447	0.789	0.977
Low quality (≤ 9)	1.12 (1.01, 1.24)	**0.054**	**0.182**	0.417	0.890	0.976
**Allele model (T *vs.* C)**						
Overall	1.05 (1.01, 1.08)	**0.086**	**0.190**	0.507	0.831	0.987
Asian	1.32 (1.20, 1.46)	**0.007**	**0.046**	**0.137**	0.368	0.769
Bladder cancer	2.92 (1.69, 5.04)	**0.001**	**0.006**	**0.035**	**0.108**	**0.187**
Hepatocellular cancer	1.54 (1.14, 2.07)	**0.006**	**0.034**	**0.117**	**0.181**	0.370
HB	1.16 (1.09, 1.23)	**0.011**	**0.064**	**0.116**	**0.192**	0.473
Low quality (≤ 9)	1.09 (1.02, 1.17)	**0.043**	**0.158**	0.438	0.799	0.986

FPRR analysis results were in bold, with a prior probability <0.2. ^a^ *P* value for significant test.

### 3.3. TSA of rs743572

**Fig 6:**
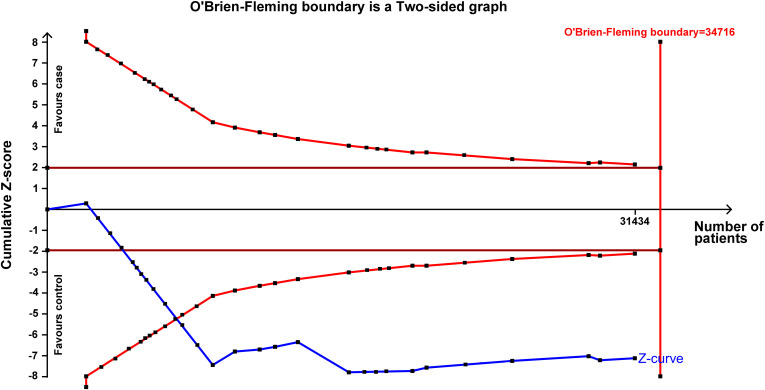
TSA of rs743572 polymorphism’s association (dominant model) with cancer risk.

As depicted in Fig 6, even though case total number did not surpass the O’Brien-Fleming boundary, the cumulative Z-curve surpassed the test sequence monitoring boundary. This observation corroborates that rs743572 is markedly linked to cancer risk.

### 3.4. Publication bias

**Fig 7:**
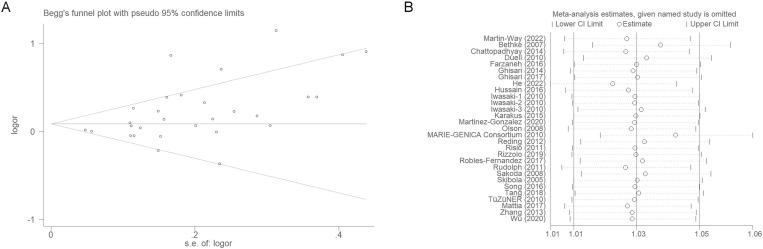
Publication bias and Sensitivity analysis.

Both Begg’s and Egger’s tests revealed no substantial publication bias this meta-analysis (Figure 7A; Table 5). Moreover, as demonstrated by sensitivity analysis, no individual study notably altered the conclusion (Figure 7B).

**Table 5:** Begg’s and Egger’s tests for publication bias.

Model	Dominant	Recessive	Homozygote	Heterozygote	Allele
P_Begg_	0.389	0.514	0.643	0.715	0.402
P_Egger_	0.257	0.482	0.379	0.843	0.371

## 4. Discussion

It is increasingly evident that genetics is pivotal in influencing cancer risk [1,4]. Given that polymorphism represents a primary source of variation in human genetic information, polymorphisms’s association with cancer is gaining much attention [3]. Advancements in medical technology have sparked significant interest in tumor genetic susceptibility, leading to a surge in research on genetic polymorphisms related to cancer. Among these, genetic polymorphisms within the cytochrome P450 family have become a pivotal factor in exploring polymorphisms associated with malignant tumor risks [15,19].

CYP17A1 is crucial for intricate cancer biology, particularly in relation to hormone-dependent cancers. CYP17A1 is a pivotal enzyme involved in steroid hormone biosynthesis, including androgens and estrogens [24,34–36]. The dysregulation of these hormones contributes to developing and facilitating diverse cancers, and CYP17A1 emerges as a crucial player in this context. CYP17A1 has been extensively explored for its role in hormone-dependent malignancies, such as prostate cancer and hormone-sensitive breast cancer [19–26]. In our study, we found a significant association between CYP17A1 rs743572 polymorphism with both hormone-sensitive and non-hormone-sensitive cancers, reflecting its dual role in steroid hormone synthesis and broader metabolic pathways. For instance, the increased risk of breast cancer supports its involvement in estrogen biosynthesis, particularly in hormone receptor-positive subtypes. Interestingly, the significant association with bladder cancer may reflect androgen-related mechanisms, as androgen signaling has been implicated in bladder tumorigenesis. These findings highlight the need for further studies stratified by hormone receptor status to clarify cancer type-specific effects.

The enzyme CYP17A1 plays a critical role in steroid hormone biosynthesis by catalyzing two key reactions, 17α-hydroxylase and 17, 20-lyase activities, which convert pregnenolone and progesterone into precursors for glucocorticoids, mineralocorticoids, and sex hormones such as androgens and estrogens [25]. This enzyme is a pivotal target in hormone-dependent cancer therapies, especially in prostate cancer and breast cancer. Prostate cancer, a prevalent malignancy in men, is often driven by androgens, the male sex hormones. Inhibition of CYP17A1 has thus become a targeted strategy to impede androgen synthesis and, consequently, hinder the growth and progression of prostate cancer. Similarly, in hormone-sensitive breast cancer, where estrogen signaling fuels tumor development, CYP17A1 has garnered attention. The enzyme contributes to the synthesis of estrogen precursors, and interventions targeting CYP17A1 have been explored to mitigate estrogen production and dampen the stimulatory effects on breast cancer cells [18,22,27]. This approach is particularly relevant in postmenopausal women, where estrogen produced by the ovaries is shifted to be produced by peripheral tissues, including the adrenal glands, where CYP17A1 is active. However, side effects from glucocorticoid depletion and genetic variability in CYP17A1 can influence treatment efficacy and patient prognosis. Selective CYP17A1 lyase inhibitors offer a promising approach to optimize therapy by reducing side effects while maintaining anti-androgen effects. Abiraterone acetate, a notable CYP17 inhibitor, has shown efficacy in treating metastatic castration-resistant prostate cancer, demonstrating the clinical significance of targeting CYP17A1 in managing advanced stages of the disease [31,33]. While the inhibition of CYP17A1 presents a promising approach in certain cancer types, it is essential to consider potential side effects and the broader impact on steroid hormone balance in the body. Hormonal imbalances induced by CYP17A1 inhibition may lead to adverse effects, underscoring the need for careful clinical management and monitoring.

This review revealed a marked association between rs743572 and elevated cancer susceptibility risk, which was validated through FPRP and TSA analyses. Stratified analyses by cancer type revealed a significant association between rs743572 and bladder cancer, breast cancer, non-Hodgkin lymphoma, and hepatocellular cancer. However, most subgroups had insufficient numbers, which may attenuate the statistical power. Nevertheless, limited data restrain the assessment of rs743572’s association with ER-negative and ER-positive breast cancer. Future research with a larger cohort is needed.

This present analysis possesses several strengths: (1) findings were robustly confirmed through TSA analysis, enhancing result reliability; (2) all incorporated studies adhered to the Hardy-Weinberg Equilibrium (HWE), contributing to the overall study reliability; (3) this systematic evaluation represents the inaugural analysis examining the relationship between CYP17A1 rs743572 and cancer risk; (4) FPRP analysis was applied to all significant observations.

Nevertheless, there are certain limitations. (1) The inclusion of subjects was restricted to Caucasians and Asians, lacking representation from other ethnic groups, potentially introducing publication bias. (2) Some subgroups exhibited a relatively small number of studies on CYP17A1 rs743572, raising the possibility of significant or insignificant outcomes occurring by chance. (3) Detailed information on factors (exposure to radiation and carcinogens, smoking, etc.) was not uniformly available in all included studies, hampering stratification analyses. (4) CYP17A1 plays an essential role in the synthesis of steroid hormones, further studies stratified by hormone receptor status to clarify cancer type-specific effects are needed. Thus, future research should aim for larger sample sizes, incorporate multi-racial populations, and adopt a standardized, multi-center approach to provide more comprehensive and detailed data.

## 5. Conclusions

In summary, this systematic meta-analysis suggests a crucial role for the rs743572 polymorphism in cancer pathogenesis, with particular prominence observed in bladder cancer, breast cancer, non-Hodgkin lymphoma, and hepatocellular cancer—a noteworthy observation supported by FPRP evaluation. However, it is essential to note that future comprehensive studies are warranted to validate and reinforce the robustness of our findings.

## Supporting information

S1 Table Score of quality assessment. (DOCX)

S1 File Original Data. (ZIP)

S2 File PRISMA_2020_checklist. (DOCX)

## References

[1] Sung H, Ferlay J, Siegel RL, Laversanne M, Soerjomataram I, Jemal A, et al. Global cancer statistics 2020: GLOBOCAN estimates of incidence and mortality worldwide for 36 cancers in 185 countries. CA Cancer J Clin. 2021;71(3):209–49. doi: 10.3322/caac.21660 33538338

[2] Andrawus M, Sharvit L, Shekhidem HA, Roichman A, Cohen HY, Atzmon G. The effects of environmental stressors on candidate aging associated genes. Exp Gerontol. 2020;137:110952. doi: 10.1016/j.exger.2020.110952 32344118

[3] Zhao M, Ma J, Li M, Zhang Y, Jiang B, Zhao X. Cytochrome P450 enzymes and drug metabolism in humans. Int J Mol Sci. 2021;22:12808. 34884615 10.3390/ijms222312808 PMC8657965

[4] Lee S-G, Kim V, Lee G-H, Kim C, Jeong E, Guengerich FP, et al. Hydroxylation and lyase reactions of steroids catalyzed by mouse cytochrome P450 17A1 (Cyp17a1). J Inorg Biochem. 2023;240:112085. doi: 10.1016/j.jinorgbio.2022.112085 36640554 PMC9892303

[5] Burris-Hiday SD, Loomis CL, Richard AM, Scott EE. Generation of human steroidogenic cytochrome P450 enzymes for structural and functional characterization. Methods Enzymol. 2023;689:3–38. doi: 10.1016/bs.mie.2023.05.010 37802575 PMC10787587

[6] Petrunak EM, Bart AG, Peng H-M, Auchus RJ, Scott EE. Human cytochrome P450 17A1 structures with metabolites of prostate cancer drug abiraterone reveal substrate-binding plasticity and a second binding site. J Biol Chem. 2023;299(3):102999. doi: 10.1016/j.jbc.2023.102999 36773804 PMC10023946

[7] Lu X, Wang L, Lin X, Huang J, Charles Gu C, He M, et al. Genome-wide association study in Chinese identifies novel loci for blood pressure and hypertension. Hum Mol Genet. 2015;24(3):865–74. doi: 10.1093/hmg/ddu478 25249183 PMC4303798

[8] Meng J, Wang S, Zhang M, Fan S, Zhang L, Liang C. TP73 G4C14-A4T14 polymorphism and cancer susceptibility: evidence from 36 case-control studies. Biosci Rep. 2018;38(6):BSR20181452. doi: 10.1042/BSR20181452 30420492 PMC6294616

[9] He J, Liao X-Y, Zhu J-H, Xue W-Q, Shen G-P, Huang S-Y, et al. Association of MTHFR C677T and A1298C polymorphisms with non-Hodgkin lymphoma susceptibility: evidence from a meta-analysis. Sci Rep. 2014;4:6159. doi: 10.1038/srep06159 25146845 PMC5381410

[10] Wacholder S, Chanock S, Garcia-Closas M, El Ghormli L, Rothman N. Assessing the probability that a positive report is false: an approach for molecular epidemiology studies. J Natl Cancer Inst. 2004;96(6):434–42. doi: 10.1093/jnci/djh075 15026468 PMC7713993

[11] He J, Wang M-Y, Qiu L-X, Zhu M-L, Shi T-Y, Zhou X-Y, et al. Genetic variations of mTORC1 genes and risk of gastric cancer in an Eastern Chinese population. Mol Carcinog. 2013;52 Suppl 1:E70-9. doi: 10.1002/mc.22013 23423739

[12] Bethke L, Webb E, Sellick G, Rudd M, Penegar S, Withey L, et al. Polymorphisms in the cytochrome P450 genes CYP1A2, CYP1B1, CYP3A4, CYP3A5, CYP11A1, CYP17A1, CYP19A1 and colorectal cancer risk. BMC Cancer. 2007;7:123. doi: 10.1186/1471-2407-7-123 17615053 PMC1925111

[13] Chattopadhyay S, Siddiqui S, Akhtar MS, Najm MZ, Deo SVS, Shukla NK, et al. Genetic polymorphisms of ESR1, ESR2, CYP17A1, and CYP19A1 and the risk of breast cancer: a case control study from North India. Tumour Biol. 2014;35(5):4517–27. doi: 10.1007/s13277-013-1594-1 24430361

[14] Duell EJ, Holly EA, Kelsey KT, Bracci PM. Genetic variation in CYP17A1 and pancreatic cancer in a population-based case-control study in the San Francisco Bay Area, California. Int J Cancer. 2010;126(3):790–5. doi: 10.1002/ijc.24792 19642097 PMC4820010

[15] Farzaneh F, Noghabaei G, Barouti E, Pouresmaili F, Jamshidi J, Fazeli A. Analysis of CYP17, CYP19 and CYP1A1 Gene Polymorphisms in Iranian Women with Breast Cancer. Asian Pac J Cancer Prev. 2016;17:23–6. 27165202 10.7314/apjcp.2016.17.s3.23

[16] Ghisari M, Eiberg H, Long M, Bonefeld-Jørgensen EC. Polymorphisms in phase I and phase II genes and breast cancer risk and relations to persistent organic pollutant exposure: a case-control study in Inuit women. Environ Health. 2014;13(1):19. doi: 10.1186/1476-069X-13-19 24629213 PMC4234380

[17] Ghisari M, Long M, Røge DM, Olsen J, Bonefeld-Jørgensen EC. Polymorphism in xenobiotic and estrogen metabolizing genes, exposure to perfluorinated compounds and subsequent breast cancer risk: A nested case-control study in the Danish National Birth Cohort. Environ Res. 2017;154:325–33. doi: 10.1016/j.envres.2017.01.020 28157646

[18] He H, Deng Y, Wan H, Shen N, Li J, Zeng Q, et al. Urinary bisphenol A and its interaction with CYP17A1 rs743572 are associated with breast cancer risk. Chemosphere. 2022;286(Pt 3):131880. doi: 10.1016/j.chemosphere.2021.131880 34426286

[19] Hussain S, Bano R, Tahir Khan M, Haroon Khan M. Association of the CYP17-34T/C Polymorphism with Pancreatic Cancer Risk. Asian Pac J Cancer Prev. 2016;17(S3):71–5. doi: 10.7314/apjcp.2016.17.s3.71 27165211

[20] Iwasaki M, Hamada GS, Nishimoto IN, Netto MM, Motola J Jr, Laginha FM, et al. Dietary isoflavone intake, polymorphisms in the CYP17, CYP19, 17beta-HSD1, and SHBG genes, and risk of breast cancer in case-control studies in Japanese, Japanese Brazilians, and non-Japanese Brazilians. Nutr Cancer. 2010;62(4):466–75. doi: 10.1080/01635580903441279 20432167

[21] Karakus N, Kara N, Ulusoy AN, Ozaslan C, Tural S, Okan I. Evaluation of CYP17A1 and LEP Gene Polymorphisms in Breast Cancer. Oncol Res Treat. 2015;38:418–22. 26407154 10.1159/000438940

[22] Martinez-Gonzalez LJ, Antúnez-Rodríguez A, Vazquez-Alonso F, Hernandez AF, Alvarez-Cubero MJ. Genetic variants in xenobiotic detoxification enzymes, antioxidant defenses and hormonal pathways as biomarkers of susceptibility to prostate cancer. Sci Total Environ. 2020;730:138314. doi: 10.1016/j.scitotenv.2020.138314 32388358

[23] Olson SH, Orlow I, Bayuga S, Sima C, Bandera EV, Pulick K, et al. Variants in hormone biosynthesis genes and risk of endometrial cancer. Cancer Causes Control. 2008;19(9):955–63. doi: 10.1007/s10552-008-9160-7 18437511 PMC2683972

[24] Martin-Way D, Puche-Sanz I, Cozar JM, Zafra-Gomez A, Gomez-Regalado MDC, Morales-Alvarez CM, et al. Genetic variants of antioxidant enzymes and environmental exposures as molecular biomarkers associated with the risk and aggressiveness of bladder cancer. Sci Total Environ. 2022;843:156965. doi: 10.1016/j.scitotenv.2022.156965 35764155

[25] The MARIE-GENICA Consortium on Genetic Susceptibility for Menopausal Hormone Therapy Related Breast Cancer Risk. Postmenopausal estrogen monotherapy-associated breast cancer risk is modified by CYP17A1_-34_T>C polymorphism. Breast Cancer Research Treatment. 2010;120:737–44. 19672705 10.1007/s10549-009-0490-2

[26] Reding KW, Chen C, Lowe K, Doody DR, Carlson CS, Chen CT, et al. Estrogen-related genes and their contribution to racial differences in breast cancer risk. Cancer Causes Control. 2012;23(5):671–81. doi: 10.1007/s10552-012-9925-x 22418777 PMC3356164

[27] Risio M, Venesio T, Kolomoets E, Armaroli P, Gallo F, Balsamo A, et al. Genetic polymorphisms of CYP17A1, vitamin D receptor and androgen receptor in Italian heredo-familial and sporadic prostate cancers. Cancer Epidemiol. 2011;35(4):e18-24. doi: 10.1016/j.canep.2010.10.003 21094112

[28] Rizzolo P, Silvestri V, Valentini V, Zelli V, Bucalo A, Zanna I. Evaluation of CYP17A1 and CYP1B1 polymorphisms in male breast cancer risk. Endocr Connect. 2019;8:1224–9. 31336362 10.1530/EC-19-0225 PMC6733362

[29] Robles-Fernandez I, Martinez-Gonzalez LJ, Pascual-Geler M, Cozar JM, Puche-Sanz I, Serrano MJ, et al. Association between polymorphisms in sex hormones synthesis and metabolism and prostate cancer aggressiveness. PLoS One. 2017;12(10):e0185447. doi: 10.1371/journal.pone.0185447 28981526 PMC5628818

[30] Rudolph A, Sainz J, Hein R, Hoffmeister M, Frank B, Försti A, et al. Modification of menopausal hormone therapy-associated colorectal cancer risk by polymorphisms in sex steroid signaling, metabolism and transport related genes. Endocr Relat Cancer. 2011;18:371–84. 21490239 10.1530/ERC-11-0057

[31] Sakoda LC, Blackston C, Doherty JA, Ray RM, Lin MG, Stalsberg H, et al. Polymorphisms in steroid hormone biosynthesis genes and risk of breast cancer and fibrocystic breast conditions in Chinese women. Cancer Epidemiol Biomarkers Prev. 2008;17(5):1066–73. doi: 10.1158/1055-9965.EPI-07-2680 18483327 PMC2791045

[32] Skibola CF, Lightfoot T, Agana L, Smith A, Rollinson S, Kao A, et al. Polymorphisms in cytochrome P450 17A1 and risk of non-Hodgkin lymphoma. Br J Haematol. 2005;129(5):618–21. doi: 10.1111/j.1365-2141.2005.05505.x 15916684

[33] Song J, Tao Z-H, Liu X-Y, Gong S, Gan L. Relationship between CYP17 gene polymorphisms and risk of prostate cancer. Genet Mol Res. 2016;15(1):15017866. doi: 10.4238/gmr.15017866 26985923

[34] Tang L, Platek ME, Yao S, Till C, Goodman PJ, Tangen CM, et al. Associations between polymorphisms in genes related to estrogen metabolism and function and prostate cancer risk: results from the Prostate Cancer Prevention Trial. Carcinogenesis. 2018;39(2):125–33. doi: 10.1093/carcin/bgx144 29228205 PMC6075364

[35] Tüzüner BM, Oztürk T, Kisakesen HI, Ilvan S, Zerrin C, Oztürk O, et al. CYP17 (T-34C) and CYP19 (Trp39Arg) polymorphisms and their cooperative effects on breast cancer susceptibility. In Vivo. 2010;24(1):71–4. 20133979

[36] De Mattia E, Cecchin E, Polesel J, Bignucolo A, Roncato R, Lupo F, et al. Genetic biomarkers for hepatocellular cancer risk in a caucasian population. World J Gastroenterol. 2017;23(36):6674–84. doi: 10.3748/wjg.v23.i36.6674 29085212 PMC5643288

[37] Zhang Y, Hua S, Zhang A, Kong X, Jiang C, Deng D, et al. Association between polymorphisms in COMT, PLCH1, and CYP17A1, and non-small-cell lung cancer risk in Chinese nonsmokers. Clin Lung Cancer. 2013;14(1):45–9. doi: 10.1016/j.cllc.2012.04.004 22658813

[38] Wu X, Xu QJ, Chen PZ, Yu CB, Ye LF, Li T. Association between CYP17A1, CYB5A polymorphisms and efficacy of abiraterone acetate/prednisone treatment in castration-resistant prostate cancer patients. Pharmgenomics Pers Med 2020;13: 181–8. 32581567 10.2147/PGPM.S245086 PMC7280245

