# Supplementary material for: Association between CYP17A1 rs743572 polymorphism and cancer risk: A meta-analysis
Source: PLoS One. 2025 Jun 25;20(6):e0326843. doi: 10.1371/journal.pone.0326843 (PMC12193074; doi:10.1371/journal.pone.0326843)
Supplement: S1 Table — (DOCX) [file pone.0326843.s001.docx]

**S1 Table. Score of quality assessment**

| **Criteria** | **Score** |
| --- | --- |
| Case representation |  |
| from population cancer registry | 2 |
| from hospital | 1 |
| Not described | 0 |
| Control representation |  |
| Population-based | 3 |
| Blood donors | 2 |
| Hospital-based | 1 |
| Not described | 0 |
| Cancer case ascertainment |  |
| Validation by histopathology | 2 |
| Patient medical record | 1 |
| Not described | 0 |
| Control selection |  |
| Matched with cases by age and sex | 2 |
| Matched with cases only by age or by sex | 1 |
| Not matched or not descried | 0 |
| Genotyping examination |  |
| Blind genotyping and quality control | 2 |
| Blind genotyping or quality control | 1 |
| No blind genotyping and quality control | 0 |
| HWE |  |
| HWE in the control group | 1 |
| HWD in the control group or not mentioned | 0 |
| Total sample size |  |
| > 1000 | 3 |
| 501 - 1000 | 2 |
| 201 - 500 | 1 |
| ≤ 200 | 0 |
