# Supplementary material for: Association between CYP17A1 rs743572 polymorphism and cancer risk: A meta-analysis
Source: PLoS One. 2025 Jun 25;20(6):e0326843. doi: 10.1371/journal.pone.0326843 (PMC12193074; doi:10.1371/journal.pone.0326843)
Supplement: Attachment — Submitted filename: Response to Reviewers.doc [file pone.0326843.s004.doc]

**ACADEMIC EDITOR:**

1.**References:** Please review and revise the list of references carefully. In particular, **Reference number 3 appears to be incomplete**. Kindly provide the full citation, including the title, journal name, volume, issue, page numbers. Additionally, for **Reference number 34**, please ensure that the **year of publication is mentioned**, along with other complete bibliographic details.

Response: Thanks for your kind help. We have rectified the reference format as you suggested, please check MS for details.

2. **Figures: Figures 7A and 7B are not clear** in the current version of the manuscript. The resolution appears low and important details are difficult to interpret. It is recommended to **replace them with high-resolution images**, ensuring all labels and legends are clearly visible.

Response: We have provided higher-resolution versions of Figures 7A and 7B with clearer labels and legends in the revised manuscript. Please check them for details. Thanks very much for your helpful suggestions.

3. **Forest Plot:** A **comment on the forest plot is necessary** to aid interpretation. Authors are encouraged to include a brief explanation in the results or discussion section about what the forest plot depicts, including the direction and strength of the effect sizes, confidence intervals, and any observed heterogeneity among the studies.
 Response: Thanks for these valuable comments. We have added the detailed information about the forest plots in the Figure legends. Please check Figures 2-5 for details.

Please submit your revised manuscript by Jun 26 2025 11:59PM. If you will need more time than this to complete your revisions, please reply to this message or contact the journal office at [plosone@plos.org](mailto:plosone@plos.org). When you're ready to submit your revision, log on to https://www.editorialmanager.com/pone/ and select the 'Submissions Needing Revision' folder to locate your manuscript file.

Please include the following items when submitting your revised manuscript:

- A rebuttal letter that responds to each point raised by the academic editor and reviewer(s). You should upload this letter as a separate file labeled 'Response to Reviewers'.
- A marked-up copy of your manuscript that highlights changes made to the original version. You should upload this as a separate file labeled 'Revised Manuscript with Track Changes'.
- An unmarked version of your revised paper without tracked changes. You should upload this as a separate file labeled 'Manuscript'.

If you would like to make changes to your financial disclosure, please include your updated statement in your cover letter. Guidelines for resubmitting your figure files are available below the reviewer comments at the end of this letter.

If applicable, we recommend that you deposit your laboratory protocols in protocols.io to enhance the reproducibility of your results. Protocols.io assigns your protocol its own identifier (DOI) so that it can be cited independently in the future. For instructions see: [https://journals.plos.org/plosone/s/submission-guidelines#loc-laboratory-protocols](https://journals.plos.org/plosone/s/submission-guidelines#loc-laboratory-protocols). Additionally, PLOS ONE offers an option for publishing peer-reviewed Lab Protocol articles, which describe protocols hosted on protocols.io. Read more information on sharing protocols at <https://plos.org/protocols?utm_medium=editorial-email&utm_source=authorletters&utm_campaign=protocols>.

We look forward to receiving your revised manuscript.

Kind regards,

Dr. Charushila Yuvraj Kadam, PhD
Academic Editor
PLOS ONE

Journal Requirements:

When submitting your revision, we need you to address these additional requirements.

1. Please ensure that your manuscript meets PLOS ONE's style requirements, including those for file naming. The PLOS ONE style templates can be found at https://journals.plos.org/plosone/s/file?id=wjVg/PLOSOne_formatting_sample_main_body.pdf and https://journals.plos.org/plosone/s/file?id=ba62/PLOSOne_formatting_sample_title_authors_affiliations.pdf

 Response: Confirmed.

2. As required by our policy on Data Availability, please ensure your manuscript or supplementary information includes the following:

A numbered table of all studies identified in the literature search, including those that were excluded from the analyses.
For every excluded study, the table should list the reason(s) for exclusion.
If any of the included studies are unpublished, include a link (URL) to the primary source or detailed information about how the content can be accessed.
A table of all data extracted from the primary research sources for the systematic review and/or meta-analysis. The table must include the following information for each study:
Name of data extractors and date of data extraction
Confirmation that the study was eligible to be included in the review.
All data extracted from each study for the reported systematic review and/or meta-analysis that would be needed to replicate your analyses.
If data or supporting information were obtained from another source (e.g. correspondence with the author of the original research article), please provide the source of data and dates on which the data/information were obtained by your research group.
If applicable for your analysis, a table showing the completed risk of bias and quality/certainty assessments for each study or outcome. Please ensure this is provided for each domain or parameter assessed. For example, if you used the Cochrane risk-of-bias tool for randomized trials, provide answers to each of the signalling questions for each study. If you used GRADE to assess certainty of evidence, provide judgements about each of the quality of evidence factor. This should be provided for each outcome.
An explanation of how missing data were handled.
This information can be included in the main text, supplementary information, or relevant data repository. Please note that providing these underlying data is a requirement for publication in this journal, and if these data are not provided your manuscript might be rejected.
Response: Thanks very much for the valuable information. We have carefully checked each item to ensure data availability; all the requirements you mentioned above are included in the current MS.

3. PLOS requires an ORCID iD for the corresponding author in Editorial Manager on papers submitted after December 6th, 2016. Please ensure that you have an ORCID iD and that it is validated in Editorial Manager. To do this, go to ‘Update my Information’ (in the upper left-hand corner of the main menu), and click on the Fetch/Validate link next to the ORCID field. This will take you to the ORCID site and allow you to create a new iD or authenticate a pre-existing iD in Editorial Manager.

 Response: Thanks. We have registered and validated the corresponding author's ORCID iD in Editorial Manager as required. The information has been successfully updated in the submission system.

 4. We noticed you have some minor occurrence of overlapping text with the following previous publication(s), which needs to be addressed:

https://portlandpress.com/bioscirep/article/39/9/BSR20190368/220428/Association-of-the-vitamin-D-metabolism-gene-GC

In your revision ensure you cite all your sources (including your own works), and quote or rephrase any duplicated text outside the methods section. Further consideration is dependent on these concerns being addressed.

Response: Thank you for bringing this to our attention. We will properly cite the reference and rephrase any overlapping text in the revised manuscript.

5. Thank you for stating the following financial disclosure:
This research was funded by grants from Chongqing Young and Middle-aged Medical High-end Talents (No. YXGD202405), Chongqing District and County Head Goose Talents, Chongqing Science and Technology and Health Joint Scientific Research Project on Traditional Chinese Medicine (No. 2024ZYYB036), and Chongqing Banan District Science and Technology and Health Joint Scientific Research Project on Traditional Chinese Medicine (No. BNWJ202300112).
Please state what role the funders took in the study. If the funders had no role, please state: "The funders had no role in study design, data collection and analysis, decision to publish, or preparation of the manuscript."
If this statement is not correct you must amend it as needed.
Please include this amended Role of Funder statement in your cover letter; we will change the online submission form on your behalf.

Response: Thanks for your kind help. We have added the detailed information about the role of funders in the MS, please check “Financial disclosure” for details.

6. In the online submission form, you indicated that the datasets generated during and/or analysed during the current study are available from the corresponding author on reasonable request.
All PLOS journals now require all data underlying the findings described in their manuscript to be freely available to other researchers, either 1. In a public repository, 2. Within the manuscript itself, or 3. Uploaded as supplementary information.
This policy applies to all data except where public deposition would breach compliance with the protocol approved by your research ethics board. If your data cannot be made publicly available for ethical or legal reasons (e.g., public availability would compromise patient privacy), please explain your reasons on resubmission and your exemption request will be escalated for approval.

 Response: Confirmed.

7. [Please remove all personal information, ensure that the data shared are in accordance with participant consent, and re-upload a fully anonymized data set.

Note: spreadsheet columns with personal information must be removed and not hidden as all hidden columns will appear in the published file.

Additional guidance on preparing raw data for publication can be found in our Data Policy (https://journals.plos.org/plosone/s/data-availability#loc-human-research-participant-data-and-other-sensitive-data) and in the following article: http://www.bmj.com/content/340/bmj.c181.long.](http://www.bmj.com/content/340/bmj.c181.long.)

Response: Confirmed.

8. We notice that your supplementary [table] are included in the manuscript file. Please remove them and upload them with the file type 'Supporting Information'. Please ensure that each Supporting Information file has a legend listed in the manuscript after the references list.

Response: Thank you for your kind advice. We have now removed the supplementary tables from the main manuscript and uploaded them as separate Supporting Information files, with corresponding legends included after the reference list. Please check it for details.

9. [Please include captions for your Supporting Information files at the end of your manuscript, and update any in-text citations to match accordingly. Please see our Supporting Information guidelines for more information: http://journals.plos.org/plosone/s/supporting-information.](http://journals.plos.org/plosone/s/supporting-information.)

 Response: Confirmed.

10. Please review your reference list to ensure that it is complete and correct. If you have cited papers that have been retracted, please include the rationale for doing so in the manuscript text, or remove these references and replace them with relevant current references. Any changes to the reference list should be mentioned in the rebuttal letter that accompanies your revised manuscript. If you need to cite a retracted article, indicate the article’s retracted status in the References list and also include a citation and full reference for the retraction notice.
 Response: Confirmed.

[Note: HTML markup is below. Please do not edit.]

Reviewers' comments:

Reviewer's Responses to Questions

**Comments to the Author**

1. Is the manuscript technically sound, and do the data support the conclusions?

The manuscript must describe a technically sound piece of scientific research with data that supports the conclusions. Experiments must have been conducted rigorously, with appropriate controls, replication, and sample sizes. The conclusions must be drawn appropriately based on the data presented.

Reviewer #1: Yes

Reviewer #2: Yes

2. Has the statistical analysis been performed appropriately and rigorously?

Reviewer #1: I Don't Know

Reviewer #2: No

3. Have the authors made all data underlying the findings in their manuscript fully available?

The [PLOS Data policy](http://www.plosone.org/static/policies.action#sharing) requires authors to make all data underlying the findings described in their manuscript fully available without restriction, with rare exception (please refer to the Data Availability Statement in the manuscript PDF file). The data should be provided as part of the manuscript or its supporting information, or deposited to a public repository. For example, in addition to summary statistics, the data points behind means, medians and variance measures should be available. If there are restrictions on publicly sharing data—e.g. participant privacy or use of data from a third party—those must be specified.

Reviewer #1: Yes

Reviewer #2: Yes

4. Is the manuscript presented in an intelligible fashion and written in standard English?

PLOS ONE does not copyedit accepted manuscripts, so the language in submitted articles must be clear, correct, and unambiguous. Any typographical or grammatical errors should be corrected at revision, so please note any specific errors here.

Reviewer #1: Yes

Reviewer #2: Yes

5. Review Comments to the Author

Please use the space provided to explain your answers to the questions above. You may also include additional comments for the author, including concerns about dual publication, research ethics, or publication ethics. (Please upload your review as an attachment if it exceeds 20,000 characters)

Reviewer #1: The meta analysis covers an important topic for cancer etiology, although few papers have issued this point but the meta analysis covered most of the publications. The methodology and materials are accepted, the figures and tables are accepted, discussion and results are accepted

Response: Thanks very much for your positive comments.

Reviewer #2: The authors have conducted an important meta analysis on the association between CYP17A1 rs743572 polymorphism and cancer susceptibility. They have presented a well written manuscript and have presented their findings appropriately. However, to improve on the manuscript the authors should address the following comments.
1. Considering that CYP17A1 is involved in the synthesis of steroid hormones, the findings should show the significance of the enzyme by comparing its impact on hormone sensitive and non-hormone sensitive cancers.

Response: We sincerely appreciate your valuable comments about the importance of differentiating the impact of the CYP17A1 rs743572 polymorphism on hormone-sensitive and non-hormone-sensitive cancers. Our meta-analysis revealed the potential associations with both cancer types, consistent with CYP17A1's dual roles in steroid hormone synthesis and broader metabolic pathways. For instance, in breast cancer, the observed risk elevation (dominant model OR=1.08, 95% CI=1.01–1.15) aligns with CYP17A1’s function in estrogen precursor synthesis, particularly in hormone receptor-positive breast cancer. Interestingly, we also noticed the unexpected associations with bladder cancer (dominant, OR=3.14, 95% CI=1.70-5.80) may reflect underlying mechanisms (androgen signaling). We acknowledge that these results are relatively preliminary, further stratification analysis by hormone receptor status is still needed for a deeper clarification. We have added the relevant contents in the Discussion section of the latest MS, please check it for more information: “In our study, we found a significant association between CYP17A1 rs743572 polymorphism with both hormone-sensitive and non-hormone-sensitive cancers, reflecting its dual role in steroid hormone synthesis and broader metabolic pathways. For instance, the increased risk of breast cancer supports its involvement in estrogen biosynthesis, particularly in hormone receptor-positive subtypes. Interestingly, the significant association with bladder cancer may reflect androgen-related mechanisms, as androgen signaling has been implicated in bladder tumorigenesis. These findings highlight the need for further studies stratified by hormone receptor status to clarify cancer type-specific effects”; “(4) CYP17A1 plays an essential role in the synthesis of steroid hormones, further studies stratified by hormone receptor status to clarify cancer type-specific effects are needed.”.

2. The effect of the enzyme on treatment using steroid and hormone therapy and how this could affect disease progression and outcome in patients.

Response: Thanks for the intriguing comments. As well accepted, CYP17A1 is essential in steroid hormone biosynthesis, and its inhibition forms the basis of steroid and hormone therapy in hormone-dependent cancers. Effective inhibition reduces androgen levels, slowing disease progression and improving outcomes in patients, particularly those with breast cancer, prostate cancer. However, side effects from glucocorticoid depletion and genetic variability in CYP17A1 can influence treatment efficacy and patient prognosis. Selective CYP17A1 lyase inhibitors offer a promising approach to optimize therapy by reducing side effects while maintaining anti-androgen effects. We have added the corresponding contents in the Discussion section to clarify this point, please check for details: “The enzyme CYP17A1 plays a critical role in steroid hormone biosynthesis by catalyzing two key reactions, 17α-hydroxylase and 17, 20-lyase activities, which convert pregnenolone and progesterone into precursors for glucocorticoids, mineralocorticoids, and sex hormones such as androgens and estrogens. This enzyme is a pivotal target in hormone-dependent cancer therapies, especially in prostate cancer and breast cancer”; “However, side effects from glucocorticoid depletion and genetic variability in CYP17A1 can influence treatment efficacy and patient prognosis. Selective CYP17A1 lyase inhibitors offer a promising approach to optimize therapy by reducing side effects while maintaining anti-androgen effects”.

6. PLOS authors have the option to publish the peer review history of their article ([what does this mean?](https://journals.plos.org/plosone/s/editorial-and-peer-review-process#loc-peer-review-history)). If published, this will include your full peer review and any attached files.

If you choose “no”, your identity will remain anonymous but your review may still be made public.

**Do you want your identity to be public for this peer review?** For information about this choice, including consent withdrawal, please see our [Privacy Policy](https://www.plos.org/privacy-policy).

Reviewer #1: **Yes:** Haytham Abdelkader

Reviewer #2: No

[NOTE: If reviewer comments were submitted as an attachment file, they will be attached to this email and accessible via the submission site. Please log into your account, locate the manuscript record, and check for the action link "View Attachments". If this link does not appear, there are no attachment files.]

While revising your submission, please upload your figure files to the Preflight Analysis and Conversion Engine (PACE) digital diagnostic tool, <https://pacev2.apexcovantage.com/>. PACE helps ensure that figures meet PLOS requirements. To use PACE, you must first register as a user. Registration is free. Then, login and navigate to the UPLOAD tab, where you will find detailed instructions on how to use the tool. If you encounter any issues or have any questions when using PACE, please email PLOS at [figures@plos.org](mailto:figures@plos.org). Please note that Supporting Information files do not need this step.
